# Application of the Combined QCM-D/LSPR Aptasensor for Penicillin G Detection

**DOI:** 10.3390/bios15100652

**Published:** 2025-10-01

**Authors:** Sandro Spagnolo, Kiran Sontakke, Lukas Dubbert, Matthias Urban, Tomas Lednicky, Andrea Csaki, Katrin Wondraczek, Wolfgang Fritzsche, Tibor Hianik

**Affiliations:** 1 Faculty of Mathematics, Physics and Informatics, Comenius University, Mlynska Dolina F1, 84248 Bratislava, Slovakiasontakke1@uniba.sk (K.S.); 2 Leibniz Institute of Photonic Technology (Leibniz-IPHT), A. Einstein Str. 9, 07745 Jena, Germany; lukas.dubbert@leibniz-ipht.de (L.D.); matthias.urban@leibniz-ipht.de (M.U.); tomas.lednicky@leibniz-ipht.de (T.L.); andrea.csaki@leibniz-ipht.de (A.C.); katrin.wondraczek@leibniz-ipht.de (K.W.); wolfgang.fritzsche@leibniz-ipht.de (W.F.)

**Keywords:** penicillin G, DNA aptamer, quartz crystal microbalance, localized surface plasmon resonance, biosensor

## Abstract

Penicillin G (PEN) is a widely used antibiotic for treating microbial infections. However, its extensive use in veterinary medicine can lead to accumulation in animal-derived products, particularly milk and meat. This highlights the urgent need for rapid and sensitive antibiotic detection methods. In this study, we employed DNA aptamers for the detection of PEN and for the analysis of aptamer specificity using a combined approach based on quartz crystal microbalance with dissipation monitoring (QCM-D) and localized surface plasmon resonance (LSPR). QCM-D measures changes in resonant frequency, Δ*f*, and dissipation, Δ*D*, while LSPR monitors wavelength shifts in the extinction spectra corresponding to changes at the surface of gold nanoparticles (AuNPs). Thiolated aptamers were chemisorbed onto the surface of AuNPs with a diameter of 80 nm. In the presence of PEN, a redshift in the extinction spectra and a decrease in resonant frequency were observed, accompanied by an increase in dissipation due to surface viscosity effects. Significant changes in both acoustic and LSPR signals were observed at PEN concentrations as low as 1 nM. The limits of detection (LOD) for PEN, determined by QCM-D (3.0 nM, or 1.05 ng/mL)) and LSPR (3.1 nM, or 1.09 ng/mL), were similar and both were lower than the maximum residue limit (MRL) for PEN established by the EU (4 ng/mL).

## 1. Introduction

Penicillin G (PEN) belongs to the β-lactam class of antibiotics and is widely used for the treatment of bacterial infections in both human and veterinary medicine. However, uncontrolled use of antibiotics in veterinary practices resulted in their infiltration into milk or meat. Consumption of such contaminated products contributes to the development of antimicrobial resistance (AMR), which poses a significant risk to human health [1,2]. The presence of antibiotics in milk also negatively impacts cheese production by inhibiting the growth of beneficial bacteria. Therefore, the development of rapid and low-cost methods for antibiotic detection, particularly for use in dairy or food laboratories, remains a critical challenge.

Currently, the analysis of antibiotic residues in food is carried out in specialized laboratories using established techniques such as gas chromatography (GC), high-performance liquid chromatography (HPLC), and mass spectroscopy (MS). Higher accuracy of detection can be achieved by combining MS with either HPLC (MS-HPLC) or GC (MS-GC) [3,4]. Microbial methods are also employed for antibiotic detection. These are based on the analysis of bacterial growth, such as that of *Bacillus stearothermophilus*, in food samples containing antibiotics. Optimized microbial methods can detect β-lactam antibiotics in milk at limits of detection (LOD) between 30 and 80 ng/mL [5], which is significantly above the maximum residue limit (MRL) for PEN established by the EU (4 µg/kg~4 ng/mL) [6]. In addition to relatively low sensitivity and specificity, microbial methods require sterile microbial laboratory conditions. Immunochemical techniques such as enzyme-linked immunosorbent assay (ELISA) are considered as a “gold standard” for detecting food contaminants. Various commercially available ELISA kits enable antibiotic detection with a LOD below 1 ng/mL [7]. However, challenges such as potential cross-reactivity of antibodies persist [8]. The use of secondary antibodies is often required to improve specificity, though this increases the overall cost of detection. A detailed overview of current antibiotic detection methods is provided in the review by Evtugyn et al. [2].

Biosensor technology offers a promising alternative to current analytical methods. A biosensor consists of a receptor immobilized on a suitable surface with antifouling properties, enabling the detection of analytes in food samples, and a transducer that converts the chemical interactions into measurable electrical, optical, or acoustic signals, that are then analyzed using appropriate instruments, such as potentiostats, spectrometers, or mass-sensitive devices.

Among the various types of receptors, DNA/RNA aptamers have attracted increasing interest. They are known also as chemical antibodies due to their development by combinatorial chemistry by SELEX (Systematic Evolution of Ligands by Exponential Enrichments). This method was first patented by Tuerk and Gold in 1990 [9]. Since then, several modifications of this method were developed, including capture SELEX, cell SELEX, magnetic bead-based SELEX, graphene oxide (GO) based SELEX, and others. Moreover, several sophisticated strategies for post-SELEX optimization of aptamer sequences have been proposed, utilizing computer simulations and molecular docking techniques (for recent review, see Tian et al. [10]). Unlike antibodies, aptamers are more stable and can be chemically modified with ligands such as thiols, amino groups, or biotin, which facilitate oriented immobilization on sensor surfaces. Due to their higher stability, DNA aptamers are generally preferred over RNA aptamers. Currently, a wide range of DNA aptamers has been developed for antibiotic detection [2] and employed in various biosensors. Most biosensors for antibiotic detection are based on electrochemical [11] or optical [12] methods. Recent achievements in this field have been summarized in several comprehensive reviews [2,11,12]. However, a common challenge often remains: the need for signal enhancement, with or without labelling, as well as the time-consuming validation of the signal.

Electro-acoustic techniques such as quartz crystal microbalance with dissipation monitoring (QCM-D) are in principle label-free. QCM-D detects changes in the resonance frequency of a piezoelectric crystal upon loading. Shifts in resonant frequency (Δ*f*) occur due to mass changes at its surface, while changes in bandwidth reflect energy dissipation (Δ*D*) of the oscillating shear waves interacting with surrounding media (liquid or viscoelastic layer). Thus, an interaction of surrounding medium with a sensing layer including adsorption from aqueous or other sample type will influence both parameters. Compared to the reference state, their shifts reflect changes in the viscoelasticity of the surface-bound layer due to interaction or mass uptake, including trapped water in the sensing layer and conformational changes. The sensitivity of mass loading in QCM-D is represented by a minimum detectable shift in frequency of about 1 Hz. It corresponds to a real mass density loading of as little as 6.9 ng/cm^2^ in the case of the piezocrystal fundamental frequency of 8 MHz used in this work [13]. Today, QCM-D technology detects several higher harmonics (overtones) in addition to the fundamental resonance frequency, thereby enhancing detection sensitivity in liquid environment despite the low molecular weight of many antibiotics. Among label-free optical techniques, localized surface plasmon resonance (LSPR) biosensing stands out [14,15,16]. Usually, this method utilizes noble metal nanostructures such as gold nanoparticles (AuNPs) which act as signal transducers. Their conduction electrons have resonant frequency that can be spectroscopically detected from extinction spectrum. LSPR is extremely surface sensitive (∼10 nm) to changes of local refractive index (RI) in the vicinity of nanoparticle surface and shift due to its changes, such as molecular binding events (e.g., aptamer–antibiotic, thiol–AuNP) [17,18,19].

The combination of LSPR and QCM method offers several advantages in analytical diagnostics due to their distinct detection principles. QCM-D analysis returns the areal mass density of bonded molecules along with information on conformational changes related to changes in adjacent medium or layer viscosity. In contrast, LSPR gives only the mass-related number of molecules. Both methods can be operated in real-time. The combination of QCM-D and LSPR usually relies on utilizing a complex, resource-intense nanostructuring of the sensor electrodes [20,21,22].

In the present study, we report on the pioneering application of a combined QCM-D and LSPR approach for the detection of PEN. It employs a simple, easy-to-carry-out protocol based on easy adsorption chemistry, a cost-efficient method for depositing colloidal nanospheres onto QCM sensor chips. This strategy capitalizes on the strengths of both techniques, thereby enhancing the precision of detection and enabling comparative analysis of the methodologies, while simultaneously reducing costs and simplifying the preparation process. The integrated QCM-D/LSPR approach enabled selective detection of the antibiotic, with comparable limits of detection (LOD) for QCM-D (3.0 nM, corresponds to 1.05 ng/mL) and LSPR (3.1 nM, corresponds to 1.09 ng/mL), both below the EU-established maximum residue limit (MRL) for PEN (4 ng/mL).

## 2. Materials and Methods

### 2.1. Chemicals

The experiments were performed using phosphate-buffered saline (PBS) composed of 10 mM Na_2_HPO_4_, 1.8 mM KH_2_PO_4_, 137 mM NaCl, and 2.7 mM KCl, pH 7.4. Deionized water (DI) with a resistivity of 18 MΩ·cm, made by a Purelab Classic UV system (Elga, High Wycombe, UK), was used for the preparation of all aqueous solutions. AuNPs with an average diameter of 80 nm, stabilized in citrate buffer, have been purchased from Sigma-Aldrich, Cat. No. 742023 (Darmstadt, Germany). 6-mercapto-1-hexanol (MCH), penicillin G sodium salt (PEN), Cat. No. 13752, and oxytetracycline hydrochloride (OTC), Cat. No. O5875, sodium dodecylsulphate (SDS), (3-aminopropyl) triethoxysilane (APTES), and DNA from salmon sperm were purchased from Sigma-Aldrich (Darmstadt, Germany). Standard chemicals including ethanol, NaCl, NH_3_, H_2_O_2_ and acetic acid were purchased from Slavus (Bratislava, Slovakia). For the preparation of the aptasensor, we used DNA aptamers specifically binding to PEN with the following sequence: 5′- HS-(CH_2_)_6_-CTG AAT TGG ATC TCT CTT CTT GAG CGA TCT CCA CA-3′ (HS-Apt-PEN) [23], synthesized by Generi Biotech (Hradec Králové, Czech Republic). Additionally, thiolated DNA aptamers specific to oxytetracycline (OTC) were used, with the sequence: 5′- HS-(CH_2_)_6_-GGG GGC ACA CAT GTA GGT GCT GTC CAG GTG TGG TTG TGG T-3′ (HS-Apt-OTC) [24], synthesized by GeneCust (Boynes, France).

### 2.2. Preparation of the Sensing Surfaces and PEN Detection

#### 2.2.1. Sensor Surface Functionalization

As a substrate for sensor preparation, we used AT-cut quartz crystals with a fundamental frequency of 8 MHz and an overall diameter of 1.37 cm. The crystal surface features a circular gold electrode with an area of 0.2 cm^2^ (Total Frequency Control Ltd., Storrington, UK). The gold electrode was used for QCM-D measurements, while the surrounding transparent quartz area was used for simultaneous LSPR measurements. The crystals were initially pre-cleaned by sonication in a 1% aqueous SDS solution for 15 min, followed by rinsing with ethanol and finally by DI water. Subsequently, a basic Piranha solution was applied at ~72 °C for a minimum of 25 min for deep cleaning, followed by thorough rinsing with DI water. This cleaning step was repeated three times, with the last two rinses carried out using ethanol and then DI water, respectively. The cleaned substrates were then dried in an oven at 100 °C for at least 10 min. If needed, this drying step can be extended overnight to continue with nanoparticle immobilization the next day. After drying, the crystals were treated in a UV ozone cleaner (Ossila B.V., Leiden, Netherlands) for 30 min to enhance surface reactivity. For LSPR measurements in transparent mode, the quartz region of the piezocrystal was modified with AuNPs using APTES [25]. APTES was dissolved in 1 mM acetic acid (1% *v*/*v*) under stirring for at least 10 min for prehydrolyzation. Immediately after preparation, functionalization was performed by immersing the piezocrystals in the APTES solution on a shaker at 10 rpm for 30–60 min. After incubation, the substrates were briefly rinsed in DI water using an ultrasonic bath for 5 s, dried under a nitrogen stream, and cured in oven at 100 °C for 10 min. The crystals were then allowed to cool in ambient air for 5 min before AuNPs immobilization.

#### 2.2.2. AuNP Immobilization

AuNPs suspensions (2 mL) were concentrated by centrifugation at 6000 rpm for 8 min at 4 °C. The supernatant (1.8 mL) was discarded, and the remaining solution was vortexed briefly. This process was repeated. Eventually, 20 mL of the commercial AuNPs suspension resulted in 200 µL of a concentrated AuNPs suspension ready for use. A 20 µL aliquot of the concentrated AuNPs suspension was dropped onto the transparent side of each crystal and incubated in a humidity chamber for 30 min to promote uniform deposition. The crystals were then gently rinsed with DI water to remove excess AuNPs, taking care not to disturb the adsorbed nanoparticle layer. The substrates were dried under a nitrogen stream and inspected under a microscope to verify uniform nanoparticle coverage. The crystals were then stored in a dust-free container until further use or used immediately.

#### 2.2.3. Aptamer Functionalization of the Sensors

Prior to DNA aptamer functionalization, the AuNPs-modified crystals were cleaned with ozone treatment for approximately 5 min. This process was also employed to eliminate any residual APTES that may have weakly adsorbed onto the gold electrodes of the QCM (through non-covalent interactions mediated by its terminal amine group) and undergo self-polymerization, interfering with aptamer immobilization. At the same time, the AuNPs immobilized on the quartz regions remain anchored, as the underlying APTES layer is less accessible to ozone and not readily degraded during the cleaning process (consistent with the observation that the nanoparticles are not removed during flow-based measurements) [26]. The cleaned crystals were then assembled into the fluidic chamber setup. The subsequent QCM-D/LSPR biosensor surface layer was prepared using a continuous flow system with a flow rate of 50 µL/min for all incubation steps. A pre-flow at 100 µL/min was used before each reagent to fill the tubing and establish steady flow. The sensor surface was first flushed with citrate buffer (CB; 0.5 M Na-citrate, pH 3), followed by stabilization at 50 µL/min. To verify proper flow channel operation, alternating injections of CB and DI water were used to confirm refractive index shifts.

Surface functionalization was initiated by flowing CB until both QCM and LSPR signals stabilized. Then, a 2 µM solution of thiol-modified aptamers in CB was injected for 120 min to immobilize the capture probes onto the gold surfaces. Unbound aptamers were removed by washing with CB for 5–10 min, followed by conditioning with PBS for at least 5 min. To reduce nonspecific binding, two passivating steps were conducted. The sensor surface was first passivated with 1 mM MCH for 20 min, followed by a 5 min wash with PBS. A second blocking step was performed using 0.1 mg/mL DNA from salmon sperm for 20 min, followed by 15 min of PBS flow to ensure complete surface equilibration. The wash times were adjusted as needed to achieve signal stabilization. The functionalized biosensor surface was then ready for analyte detection.

#### 2.2.4. Biosensing

**Figure 1:**
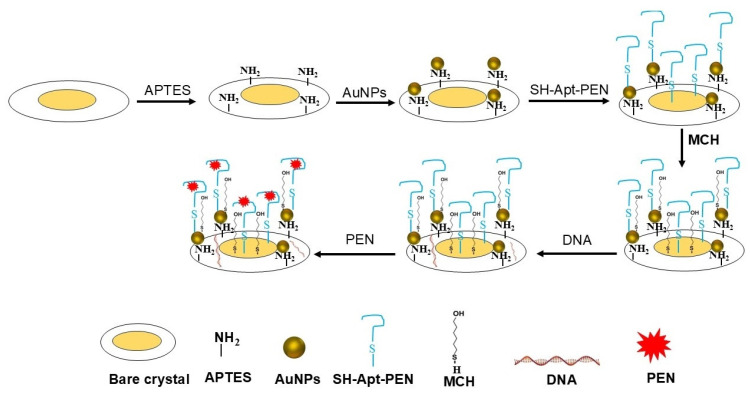
Scheme of the biosensing layer preparation, and the subsequent detection of penicillin G (PEN). The process involves APTES-mediated functionalization of the quartz crystal sensor, immobilization of gold nanoparticles (AuNPs), attachment of thiolated DNA aptamers specific to PEN, and surface passivation using 6-mercapto-1-hexanol (MCH) and salmon sperm DNA to minimize nonspecific binding. The resulting functionalized surface was employed for combined QCM-D and LSPR-based detection of PEN.

PEN solutions prepared in PBS at various concentrations were injected in flow mode over aptamer-functionalized surface for 15 min, followed by a 5 min wash with PBS. Figure 1 shows a scheme of the sensing layer preparation on the surface of the crystal.

### 2.3. Experimental Apparatus and Data Analysis

The combined QCM-D/LSPR setup consisted of the modified quartz crystal sensor inside a flow chamber with both QCM-D analysis and spectroscopic measurement. A schematic representation of the experimental setup is shown in Figure S1 of the Supplementary Material. The as-prepared QCM sensor crystal was mounted in a custom-made acrylic flow cell which included inlet and outlet ports to facilitate continuous liquid flow. A Genie Plus syringe pump (Kent Scientific, Torrington, CT, USA) was used to ensure controlled flow.

The electrodes were connected by metal contacts on the backside of the QCM crystal. The QCM-D part consisted of a computer-controlled vector impedance analyzer Sark 110 (Seeed Studio, Shenzhen, China), which enabled measurements of the impedance spectra of the oscillating quartz crystal sensor. This allowed for determination of the changes of the resonant frequency (Δ*f*) (shift of maximum of the impedance curve) and dissipation (Δ*D*) (broadening of the impedance curve), respectively, across various overtones. More precisely, due to the electromechanical coupling, the fitting of the complex impedance by the Butterworth–Van Dyke equivalent circuit allows the determination of static and motional parameters of the oscillating quartz crystal [27,28].

LSPR measurements were performed using an Ocean Optics Flame fiber optic spectrometer (Ocean Optics Inc., Dunedin, FL, USA) equipped with a 20 µm entrance slit, allowing wavelength shift (Δ*λ*) detection with a precision better than 0.1 nm. A tungsten halogen lamp (HL-2000, Ocean Optics Inc.) was used as the light source. The sample was illuminated, and the signal was collected via Ocean Optics optical fibers (OCOP400-1-SR, Ocean Optics Inc.). A Python script (version 3.13.6) was used for simultaneous control and automatized analysis of acoustic and optical measurements [29].

## 3. Results and Discussion

In all experiments, we monitored the chemisorption of DNA aptamers onto the surface of the piezocrystal coated with AuNPs (the characterization of the sensor chip by atomic force microscopy (AFM) is provided in Supplementary Material, Figure S2) by recording changes in the wavelength (Δ*λ*) using the LSPR method, and in the resonant frequency (Δ*f*) and dissipation (Δ*D*) using the QCM-D method.

**Figure 2:**
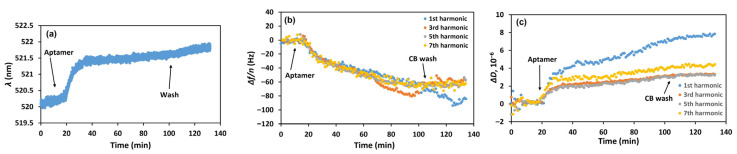
Kinetics of the changes in (**a**) LSPR wavelength (*λ*), (**b**) the resonant frequency normalized by the corresponding harmonic number (Δ*f*/*n*), and (**c**) dissipation factor (Δ*D*) following the addition of 2 µM of thiolated DNA aptamer specific to PEN (SH-Apt-PEN) and subsequent washing of the surface with CB. Arrows indicate the time points of aptamer injection and CB washing. The harmonic numbers are indicated in the inset.

**Figure 3:**
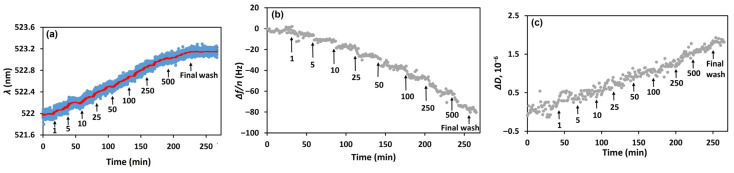
Kinetics of the changes in (**a**) LSPR wavelength (*λ*), (**b**) the 3rd harmonic of the resonant frequency normalized by the harmonic number (Δ*f*/*n*), and (**c**) the dissipation (Δ*D*), following the addition of PEN at concentrations ranging from 1 to 500 nM to the aptamer-functionalized sensing surface (SH-Apt-PEN). Arrows indicate the time points of PEN injections. After each addition and stabilization of the signal, the surface was rinsed with PBS buffer; for clarity, only the final PBS wash is marked. The red curve in panel (**a**) represents a smoothed trend of the experimental data. All PEN concentrations are expressed in nM.

In the first series of experiments, we investigated the kinetics of DNA aptamer chemisorption on the modified sensor crystal. The QCM-D and LSPR experiments were performed simultaneously using the combined cell. The introduction of thiolated DNA aptamers specific to PEN (SH-Apt-PEN) at a flow rate of 50 µL/min caused a red shift in the LSPR wavelength from 520.3 nm to 522.0 nm (see Figure 2a and Figure S3 for LSPR spectra). This sharp initial increase in wavelength was followed by a gradual stabilization over approximately 100 min. Simultaneous QCM-D measurements were conducted to monitor changes in the fundamental, 3rd, 5th, and 7th harmonic frequencies and the corresponding dissipation values (see Figure 2b and Figure 2c, respectively). Apparently, aptamer addition caused a decrease in the fundamental frequency and those of its harmonics (normalized by overtone number), clearly indicating successful chemisorption of aptamers onto the gold surface of the QCM-D sensor (cf. Figure 2b). Concurrently, an increase in dissipation was observed (Figure 3c), which we attribute to higher surface viscosity due to the interaction of the nucleotide chains with the buffer solution.

The sensing surface was then washed with citrate buffer (CB), the solvent used for aptamer preparation [30]. Only negligible shifts of the acoustic and optical values were observed. This suggests successful chemisorption of the aptamers onto the gold surface with negligible contribution from physical adsorption. Therefore, the observed decrease in resonant frequency can be attributed to both an increase in mass of the crystal surface and enhanced interfacial viscosity due to the binding interactions.

However, subsequent washing of the sensing surface with PBS caused a substantial increase in resonance frequency (approx. 70 Hz for 3rd harmonic) and slight increase in the wavelength (approx. 0.5 nm) (Figure S4 of the Supplementary Material), which is due to the effect of the changes of the ionic strength on the surface properties of the sensing layer [31,32], as well as due to influence on the refractive index in the case of LSPR [33]. After stabilization of the signal, 1 mM MCH was injected, followed by PBS washing and the addition of DNA from salmon sperm (0.1 mg/mL in PBS) to passivate the surface. These steps did not produce significant changes in the resonant frequency and wavelength (Figure S4).

Once the optical and acoustic signals were stabilized, PEN solutions at varying concentrations (1–500 nM) were introduced into the flow cell, and the kinetics of changes in the LSPR wavelength and QCM-D parameters were recorded. After each PEN injection and signal stabilization, the surface was rinsed with PBS to remove unbound analyte. Figure 3 presents the resulting kinetic profiles: (a) the LSPR wavelength shift (Δ*λ*), (b) the normalized 3rd harmonic frequency shift (Δ*f_n_*/*n*), and (c) the dissipation change (Δ*D*). Addition of PEN led to a red shift in the wavelength, a decrease in resonant frequency, and an increase in dissipation, confirming specific binding of PEN to the immobilized aptamers.

In principle, the viscosity-related contribution resulting from aptamer and PEN binding events can be evaluated by inspecting the ratio Δ*D*/Δ*f*. For rigid films, where the Sauerbrey Equation (1) [34] can be applied to estimate mass changes, this ratio should be below 10^−8^ Hz^−1^ [35]. When Δ*D*/Δ*f* ratio falls in the range of 1–4 × 10^−7^ Hz^−1^, the film is considered slightly viscoelastic. If the ratio exceeds 4 × 10^−7^ Hz^−1^, the film is classified as clearly viscoelastic [36].

(1) $$∆f=-\frac{2nf_{0}^{2}}{\sqrt{\rho_{q}{µ}_{q}}}\frac{∆m}{A}$$

where Δ*f* (Hz) is the observed frequency shift at the overtone *n*, *f*_0_ is the fundamental frequency, Δ*m* is the adsorbed mass, *A* is the active area of the quartz electrode, *μ_q_* is the shear modulus of quartz (2.947 × 10^10^ Pa), and *ρₛ* is the density of quartz (2.648 × 10^3^ kg·m^−3^) [34].

Our results showed that the Δ*D*/Δ*f* ratio following aptamer immobilization was 3.4 × 10^−7^ Hz^−1^, indicating that the sensing layer is slightly viscoelastic. After the addition of PEN at a concentration of 500 nM, this ratio increased to 9.8 × 10^−7^ Hz^−1^, providing clear evidence of increased viscosity contributions. This suggests that binding PEN to the aptamers may induce conformational changes in the oligonucleotide chains, thereby influencing the shear viscosity of the layer. This is a behavior well-described in literature for some aptamers [37]. Thus, both aptamer immobilization and PEN binding resulted in Δ*D*/Δ*f* ratios exceeding the critical threshold, meaning that the observed frequency shifts are significantly affected by viscosity contributions (i.e., increased dissipation). Nevertheless, a rough estimation of the mass density change can still be obtained using Equation (1). In this case, the surface mass density *σ* was calculated using the following equation:

σ = (Δ*m* × *N_A_*)/(*A* × *M_w_*) (2)

where *N_A_* is Avogadro’s number (6.023 × 10^23^ mol^−1^) and *M_w_* is the molecular weight of the corresponding species (aptamer: 10,758.94 Da; PEN: 334.4 Da). Based on this, the calculated surface density for the immobilized aptamers is 3.73 × 10^13^ aptamers/cm^2^, and for PEN at 500 nM, it is 2.14 × 10^14^ PEN/cm^2^. Therefore, the binding stoichiometry PEN/aptamer is approximately 5.7. Although this may represent an overestimation, it is likely that there is more than 1 PEN molecule per aptamer. Most probably also certain non-specific binding of PEN to the sensing surface occurred.

**Figure 4:**
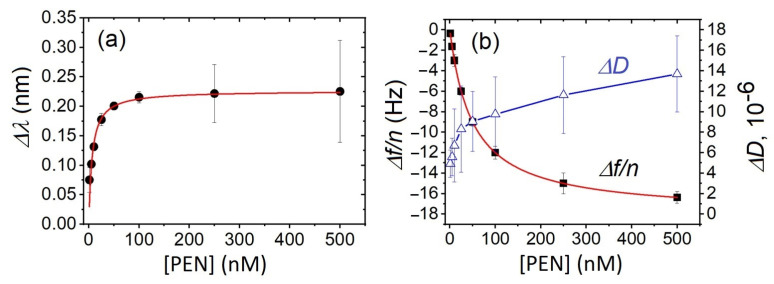
The plot of (**a**) the change in wavelength (Δ*λ*), and (**b**) the changes in the 3rd harmonic of the resonant frequency normalized by the harmonic number (Δ*f*/*n*), and the dissipation coefficient (Δ*D*) as a function of PEN concentration. The data represents the mean ± standard deviation (SD) from three independent experiments performed for each PEN concentration. Red lines are the fit according to the Langmuir isotherm (Equations (3) and (4)).

Based on the variations in both optical and acoustic responses, we constructed curves plotting the changes in the LSPR wavelength (Figure 4a) and acoustic parameters (Figure 4b) as functions of PEN concentration. As shown, both optical and acoustic signals exhibited a similar overall trend with significant changes happening in range of lower PEN concentrations (<100 nM). However, notable differences were observed beyond this concentration. Specifically, the LSPR wavelength shift reaches a plateau, indicating a possible saturation of available aptamer binding sites. In contrast, the acoustic response continued to increase up to 500 nM without reaching a clear plateau, suggesting that the QCM-D signal remains sensitive to additional mass beyond the PEN concentration at which the optical response reaches saturation.

Based on the calibration curves, the limit of detection (LOD) and limit of quantification (LOQ) for PEN were estimated using the standard criterion for LOD according to IUPAC: LOD = 3*σ*/*b*, where *σ* is the standard deviation of the baseline and *b* is the slope of the fitting equation. The LODs for QCM-D and LSPR were determined as 3.0 nM (1.05 ng/mL) and 3.1 nM (1.09 ng/mL), respectively. The corresponding LOQ values, calculated as LOQ = 10 × LOD/3.3 [38], were 9.1 nM (3.2 ng/mL) and 9.4 nM (3.3 ng/mL), respectively, both of which are below the MRL for PEN in milk (4 ng/mL).

It is noteworthy that our work is the first one that employed a combined QCM-D/LSPR method for antibiotic detection. Regarding individual techniques, QCM-D method has not yet been applied for detection PEN using aptamers.

Nevertheless, PEN and ampicillin have previously been detected using an acoustic immunosensor based on specific antibodies immobilized on a polypyrrole film, obtained by electropolymerization, and activated by glutaraldehyde [39]. The sensor achieved a LOD of 2.4 nM for PEN and 11.2 nM for ampicillin, which is comparable to the sensitivity of our combined QCM-D/LSPR aptasensor. A similar level of sensitivity in PEN detection has been achieved also by using an antibody-based surface acoustic wave (SAW) biosensor, with LOD values of 2 ng/mL in buffer and 2.2 ng/mL in low-fat milk [6].

Among plasmon methods, LSPR in transmission mode has been employed for aptamer-based detection of the antibiotic tobramycin [40]. In that study, thiolated DNA aptamers were chemisorbed onto gold nanoislands (NIs), enabling detection of tobramycin with a LOD of 0.5 µM in buffer and 3.4 µM in blood. Additionally, Blidar et al. [41] utilized a combined electrochemical surface plasmon resonance (EC-SPR) method for detecting ampicillin, reporting a LOD of 1 µM, which is significantly less sensitive than our LSPR-based results.

To further analyze the data obtained from the experiments, we applied the Langmuir approach [42] to model the adsorption of the PEN onto the sensing layer. The Langmuir model assumes the adsorption of the molecules on the surface independently without interactions between them. According to this model, the data for changes in wavelength and normalized frequency can be expressed as follows:

(3) $$\Delta \lambda =\frac{{\Delta \lambda }_{max}\;C}{K_{D}+C}$$

(4) $$\Delta f/n=\frac{{\left(\frac{\Delta f}{n}\right)}_{max}\;C}{K_{D}+C}$$

where Δ*λ_max_* and (Δ*f*/*n*)*_max_* are the maximal changes in wavelength and normalized frequency, respectively, *K_D_* is the constant of dissociation related to the affinity of PEN to the sensing layer, *C* is the PEN concentration. Figure 4 shows that both the LSPR and QCM-D data are perfectly fitted by the Langmuir equation, with a correlation coefficient R^2^ = 0.999 for both fits; Δ*λ_max_* = 0.25 nm, (Δ*f*/*n*)*_max_* = −18.0 Hz. The *K_D_* value was lower for the LSPR data: 20 nM compared to that for frequency changes: 50 nM.

The dissociation constant is related to the stability of the complex between PEN and the DNA aptamer. Lower *K_D_* values indicate higher complex stability and better affinity. We can speculate that different *K_D_* values for optical and acoustic data are related to the different surface properties for aptamer immobilization.

In the case of LSPR, aptamers are chemisorbed onto the surface of AuNPs, whereas in the acoustic experiments, they are similarly immobilized on the area of gold electrode of the piezocrystal. It is likely that the aptamers immobilized on AuNPs are characterized by more appropriate conformational flexibility, allowing better access for PEN to the binding sites.

To our knowledge, the *K_D_* value for a PEN-sensitive aptasensor has previously been reported in the paper by Guan et al. [43], in which the electrochemical detection of PEN was reported. In their study, however, a different aptamer sequence was used. The tetrahedral nanostructures that involved DNA aptamers were chemisorbed onto the surface of AuNPs attached to a reduced graphene oxide layer on a carbon screen printed electrode. The reported *K_D_* = 105.15 nM value was higher than the one observed in our work. However, their sensor demonstrated high sensitivity, with a LOD of 0.05 nM. Most recently the *K_D_* value (8.2 nM) for electrochemical PEN specific aptasensor has been determined in paper by Hu et al. [44], but with different aptamer sequence than those used in our work. Much higher value of *K_D_* (383.4 nM) has been determined earlier using another PEN sensitive aptamer and by application of fluorescence method of detection [45] (see Table S1 of Supplementary Material).

**Figure 5:**
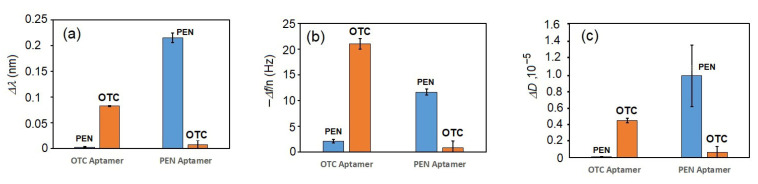
The changes in (**a**) the wavelength shift, Δ*λ*, (**b**) the 3rd harmonic of the resonant frequency normalized by the harmonic number, Δ*f*/*n*, and (**c**) the coefficient of dissipation, Δ*D*, following incubation of the aptasensors functionalized with PEN-specific and OTC-specific aptamers in the presence of 100 nM of the respective antibiotics. The results are presented as mean ± standard deviation (SD) obtained from three independent experiments for each concentration of PEN.

To evaluate the selectivity of the PEN aptamer, we analyzed the kinetic changes in both optical and acoustic responses following addition of either PEN or OTC to the surfaces functionalized with the respective aptamers. A summary of the specificity analysis for PEN and OTC at a concentration of 100 nM is presented in Figure 5. As shown in the column diagrams, the aptasensors demonstrated good selectivity for their corresponding targets, with minimal cross-reactivity.

Comparative analysis of the basic characteristics of aptamer-based biosensors for penicillin G (PEN) detection is summarized in Table S1 of the Supplementary Material. Since the first aptasensor for PEN detection has been reported by Zhao et al. [23], most of the subsequent sensors have focused on electrochemical detection [46,47,48,49,50,51,52,53,54,55]. Only a few optical approaches, primarily fluorescence- and colorimetry-based, have been explored [45,56]. So far, mass sensitive detection (QCM-D) and localized surface plasmon-based (LSPR) methods have been reported here for the first time. As shown in Table S1, a variety of aptamers were used, as well as various detection strategies with numerous detection probes, or label-free sensors were reported. Except for colorimetric method, all reported aptasensors revealed sensitivity below the MRL (4 µg/kg, corresponding to 4 ng/mL) established by the EU for milk. The highest sensitivity, with LOD of 1.04 fg/mL, was achieved by an electrochemical aptasensor employing hydrogen-bonded organic frameworks (HOFs) [55]. A similarly highly sensitive (LOD = 3.48 fg/mL) was also reported for a multimode aptasensor [57]; however, this approach requires magnetic beads separation, which complicates the procedure despite its advantages of high sensitivity and specificity. In comparison, the dual-mode aptasensor based on QCM-D/LSPR presented in this work combines the benefits of label-free detection with the ability to distinguish between mass and viscosity contributions, while still providing sufficient sensitivity. Moreover, this platform is not only suitable for PEN detecting but also serves as valuable tool for studying analyte-aptamer interaction mechanisms. As mentioned above, gravimetric sensors using specific antibodies for PEN detection have been reported previously [6,39], achieving sensitivity comparable to that of QCM-D based aptasensor. Significantly higher sensitivity of PEN detection, with an LOD of 0.27 fg/mL, was achieved by an electrochemical immunosensor in which PEN-specific antibodies were immobilized on a supported lipid bilayer (sBLM) doped with AuNPs [58]. However, the main drawback of this approach is the limited stability of sBLM-based sensors due to the presence of phospholipids. Electrochemical immunosensors for antibiotic detection have also been reviewed in detail by Patil et al. [59]. By contrast, optical immunosensor such as those employing surface plasmon resonance (SPR) have shown lower sensitivity for β-lactam antibiotics. For example, the reported LOD for ampicillin was 1 mM (350 mg/L), far above the MRL (4 ng/mL) [60]. In addition, antibody-based assays are limited by the high cost of monoclonal antibodies and their lower stability compared to DNA aptamers. It should be noted that molecularly imprinted polymers (MIP) have been applied for PEN detection, too. In this approach, the polymer is engineered to form cavities complementary to the target analyte. Incubation with the analyte leads to its adsorption into these cavities, altering the material’s physical properties such as conductivity or fluorescence. For instance, PEN has been detected using a mesoporous imprinted polymer modified by carbon dots, where fluorescence changes enable detection with a LOD of 0.12 ng/mL and good selectivity [61]. This approach needs not the aptamers or antibodies, but the surface preparation requires specific conditions.

## 4. Conclusions

We report a novel approach for the detection of the antibiotic penicillin G (PEN) using a combined QCM-D/LSPR aptasensor. The key advantage of this approach is its label-free detection capability, using DNA aptamers specific to PEN that were chemisorbed onto both gold nanoparticles (LSPR) and a thin gold layer on a piezocrystal (QCM-D). We demonstrated that the combined aptasensor exhibited a significant response at PEN concentration as low as of 1 nM. The limits of detection (LOD) for QCM-D and LSPR were similar and determined to be 3.0 nM (1.05 ng/mL) and 3.1 nM (1.09 ng/mL), respectively. Given that the maximum residue limit (MRL) for PEN contamination established by the European Union is 4 µg/kg, which corresponds to 4 ng/mL, the sensor offers adequate sensitivity. We also analyzed the binding properties of PEN to the aptamer by using the Langmuir model. We have shown that the constant of dissociation *K_D_* was lower in the optical detection (20 nM) compared to that in the acoustic determination (50 nM). This difference may be attributed to different conformational flexibility of the DNA aptamers, which are chemisorbed onto AuNPs in the LSPR setup and onto the flat gold layer in the QCM-D system. A comparative analysis of PEN and oxytetracycline (OTC) detection confirmed the good specificity of the sensor. Overall, this work serves as a proof-of-concept study demonstrating the feasibility of a combined QCM-D/LSPR method for the detection of antibiotics. Employing simultaneous measurement of two independent techniques with an easy-to-prepare sensor surface is of great advantage over conventional approaches.

## Data Availability

The data will be available upon request.

## Supplementary Materials

The following supporting information can be downloaded at: https://www.mdpi.com/article/10.3390/bios15100652/s1, S1. Setup of the apparatus for dual mode aptasensor. S2. Characterization of the sensing surface by atomic force microscopy. S3. The LSPR measurements. S4. The kinetics of the changes of the LSPR wavelength and the resonant frequency of the QCM crystal following various modifications. S5. Comparison of the sensitivity of the aptamer-based biosensors for detection of penicillin. References [23,25,43,44,46,47,48,49,50,51,52,53,54,56], are cited in Supplementary Materials.
