# Supplementary material for: Application of the Combined QCM-D/LSPR Aptasensor for Penicillin G Detection
Source: Biosensors (Basel). 2025 Oct 1;15(10):652. doi: 10.3390/bios15100652 (PMC12564596; doi:10.3390/bios15100652)
Supplement: Supplementary file 1 [file biosensors-15-00652-s001.zip › biosensors-3857551-supplementary.pdf]

biosensors

MDPI

Article

Application of the Combined QCM-D/LSPR Aptasensor for Penicillin G Detection

Sandro Spagnolo [1], Kiran Sontakke [1], Lukas Dubbert [2], Matthias Urban [2], Tomas Lednicky [2], Andrea Csaki [2], Katrin Wondraczek [2], Wolfgang Fritzsche [2] and Tibor Hianik [1,*]

[1] Faculty of Mathematics, Physics and Informatics, Comenius University, Mlynska Dolina F1, 84248 Bratislava, Slovakia; spagnolo2@uniba.sk (S.S.); sontakke1@uniba.sk (K.S.)
[2] Leibniz Institute of Photonic Technology (Leibniz-IPHT), A. Einstein Str. 9, 07745 Jena, Germany; lukas.dubbert@leibniz-ipht.de (L.D.); matthias.urban@leibniz-ipht.de (M.U.); tomas.lednicky@leibniz-ipht.de (T.L.); andrea.csaki@leibniz-ipht.de (A.C.); wolfgang.fritzsche@leibniz-ipht.de (W.F.)
* Correspondence: tibor.hianik@fmph.uniba.sk

S1. Setup of the apparatus for dual mode aptasensor

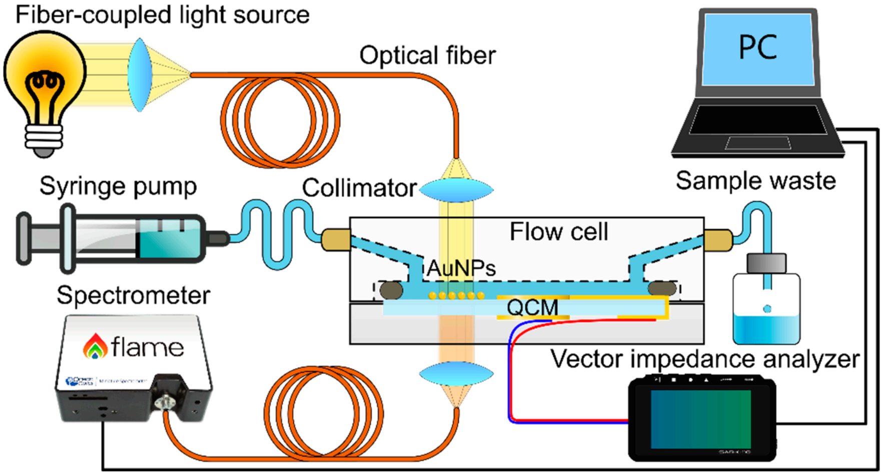

Figure S1. The scheme of the apparatus for simultaneous measurements of the acoustics (QCM-D) and optical (LSPR) parameters. The QCM crystal was mounted in a custom-made acrylic flow cell (volume 100 µL) to enable controlled liquid exchange. A syringe pump (Genie Plus, Kent Scientific, Torrington, CT, USA) was used to maintain controlled flow. Electrical connection to QCM electrodes was established via metal contacts on the backside of the crystal. The QCM-D measurements were performed using a computer-controlled vector impedance analyzer (Sark 110, Seeed Studio, Shenzhen, China), which provided impedance spectra of the oscillating quartz crystal. From these spectra, changes in resonant frequency (Δf, corresponding to the shift of the impedance maximum) and dissipation (ΔD, corresponding to the broadening of the impedance peak) were determined for multiple overtones. Simultaneously, the LSPR spectra were recorded using a broadband light source (HL-2000) and VIS spectrometer (Flame, Ocean Optics Inc., Dunedin, FL, USA).

S2. Characterization of the sensing surface by atomic force microscopy

Atomic force miscroscopy (AFM) was employed for detailed characterisation of the sensing surface. Measurements were performed in tapping mode using an NT-MDT Solver SPM microscope (Zelenograd, Moscow Region, Russian Federation). A silicon AFM cantilever (model HA-NC, NT-MDT) with a nominal spring constant of 12 N/m and a tip curvature radius of 10 nm was used. The imaging scan speed is set to 256 data

 https://doi.org/10.3390/bios15100652

points/line/second. Data analysis was carried out with the open-source software Gwyddion (http://gwyddion.net). AFM images of the bare QCM crystal, as well as of crystal with adsorbed AuNPs, DNA aptamers, and salmon sperm DNA, are presented in Figure S2. The upper part of Fig. S2 shows the location of the measured area on the QCM crystal. Images were collected from both the gold electrode (the circular spot in the center of the crystal) and the transparent region. The gold electrode served for chemisorption of the aptamers in QCM-D measurements, while the transparent region was used for AuNPs immobilization and subsequent aptamer chemisorption in LSPR experiments.

As shown in Figure S2, both the bare gold layer and transparent quartz region exhibit relatively smooth surface (a,d). Nanoparticle immobilization occurred only on the transparent region (e), although slight changes in roughness of the gold layers were also observed, likely due to chemical modification with APTES. The AuNPs were randomly distributed across the surface, and in some areas, aggregation was apparent. Futher modification with aptamers and salmon sperm DNA did not significantly affect the RMS roughness values. From the images of the transparent region, the surface density of AuNPs was estimated at ~31 NPs/μm$^{2.}$ This density is higher than in our previous report (16 NPs/μm^2) [1], which can be attributed to the use of a higher AuNP concentrations in the present work.

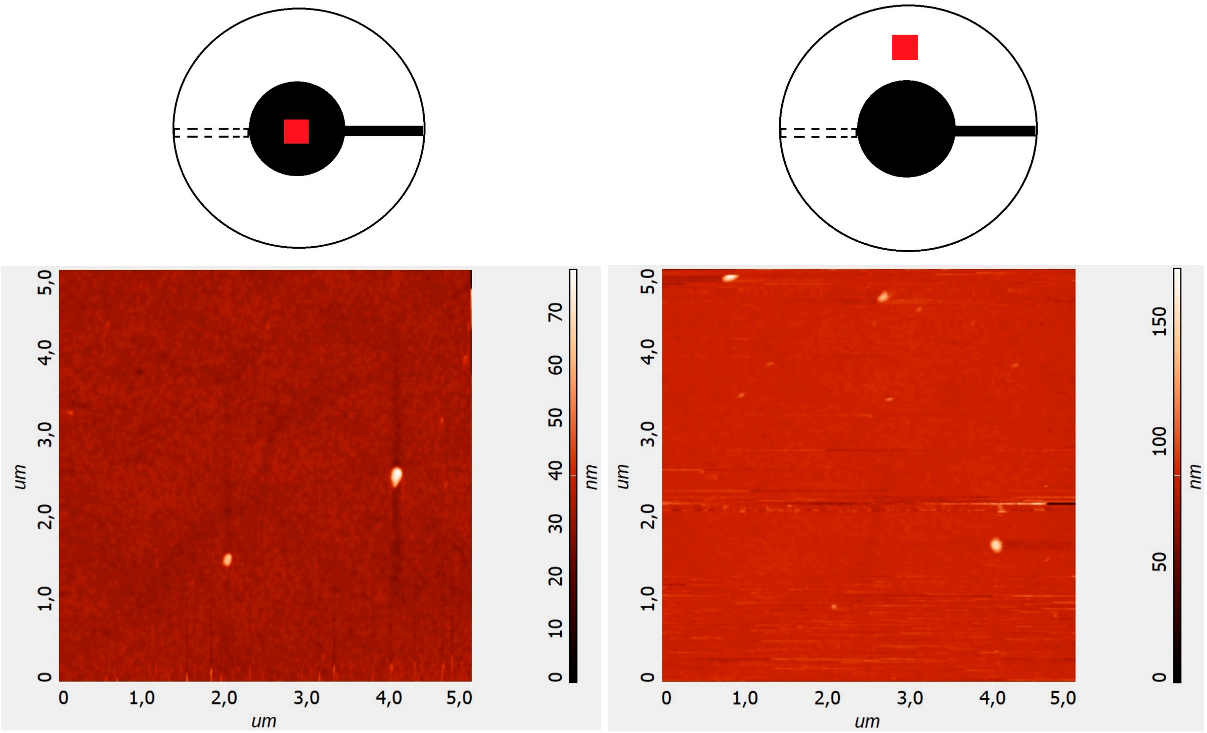

(a) RMS= 0.20 nm **(d)** RMS = 0.15 nm

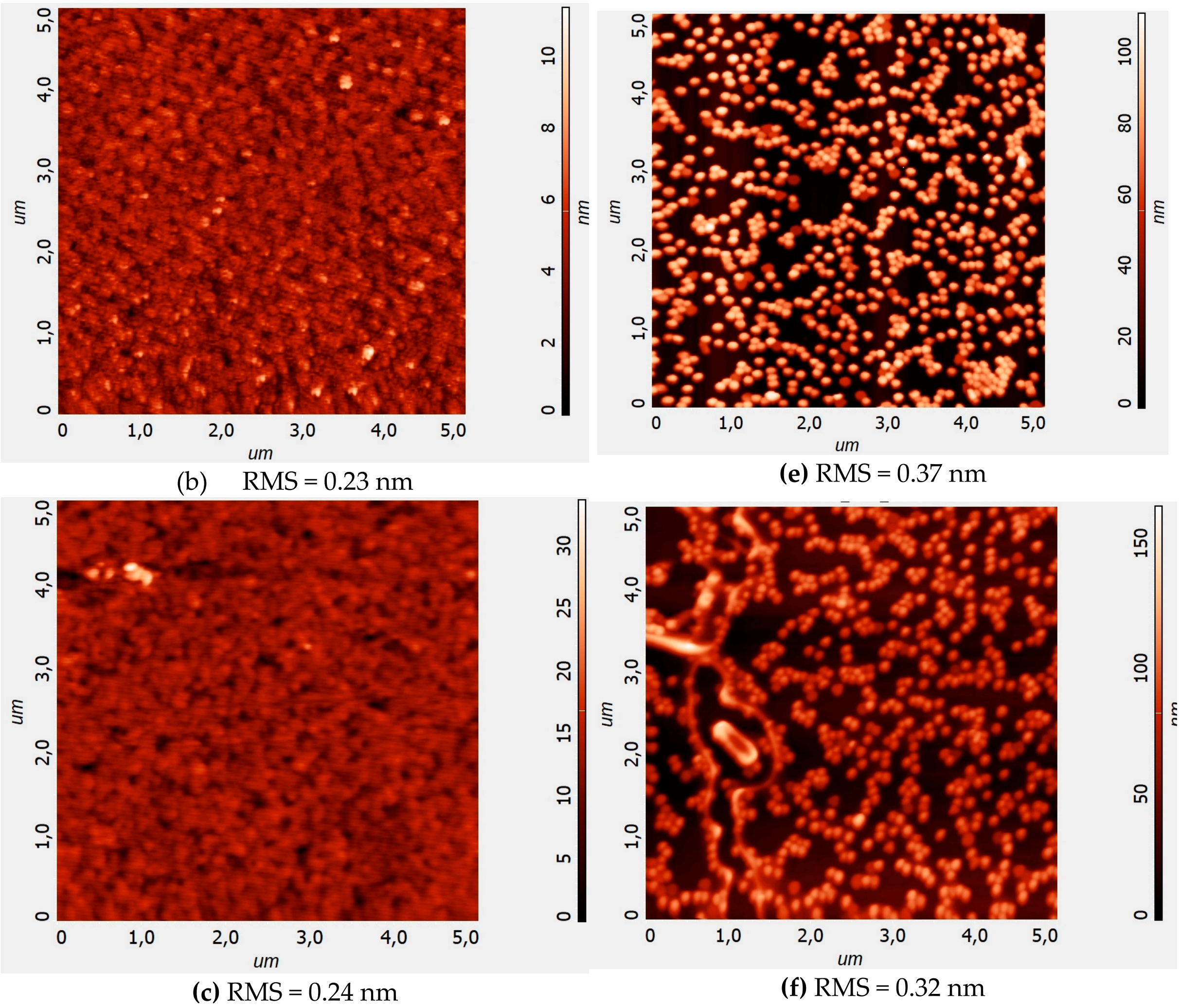

Figure S2. Schematic of the QCM crystal and AFM images of the surfaces**.** *Upper panel*: schematic of the QCM crystal illustrating the position (red square, not to scale) of scanned areas (5 µm × 5 µm) on the central gold electrod (left) used for QCM-D measurements and the transparent region (right) used for LSPR measurements. *Left panel*: AFM images of the gold electrode: (a) bare surface, (b) after incubation with APTES, and (c) after chemisorption of aptamers followed by adsorption of salmon sperm DNA. *Right panel*: AFM images of the transparent region: (d) bare surface, (e) after incubation with APTES and AuNPs, and (f) after chemisorption of aptamers and adsorption of salmon sperm DNA. Root mean square (RMS) roughness values were determined using Gwyddion software.

S3. The LSPR measurements

The LSPR measurements were performed using fiber setup illustrated in Fig. S1. The broadband light were fiber coupled from the light source and collimated as free beam with diameter of ~2 mm through the flow cell. On the other side, a collimator was used to couple transmitted light to fiber and to the spectrometer. The reference spectra were taken firstly from a blank transparent surface (without AuNPs). The extinction (LSPR) spectra were recorded in sync with pump operation and QCM-D recording. This allowed determination of the shift in LSPR band wavelength, $\Delta\lambda$, corresponding to various modification of the sensing surface. Selected spectra are shown in Figure S3, showing expected redshift of the LSPR band after 100 nM PEN incubation of the sensor.

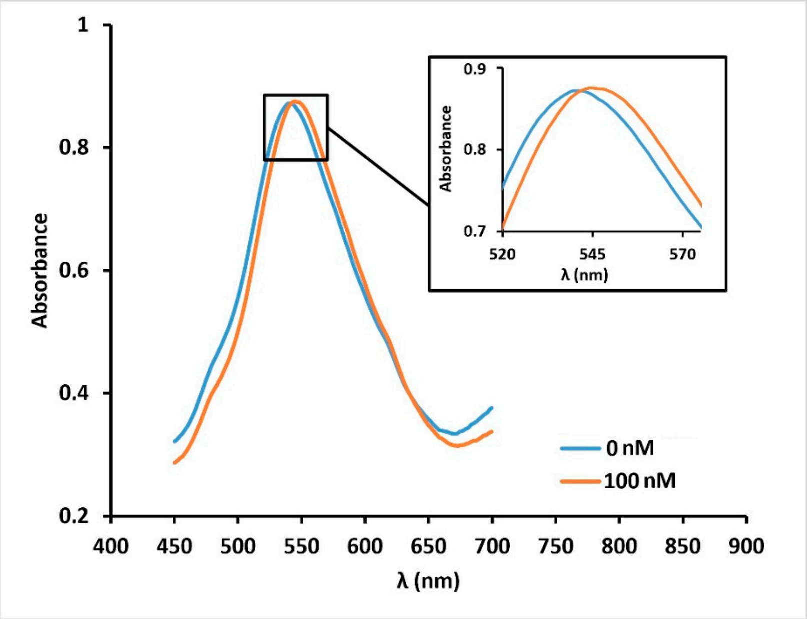

Figure S3. The LSPR spectra of the biosensor without penicillin and those incubated with 100 nM of penicillin.

S4. The kinetics of the changes of the LSPR wavelength and the resonant frequency of the QCM crystal following various modifications

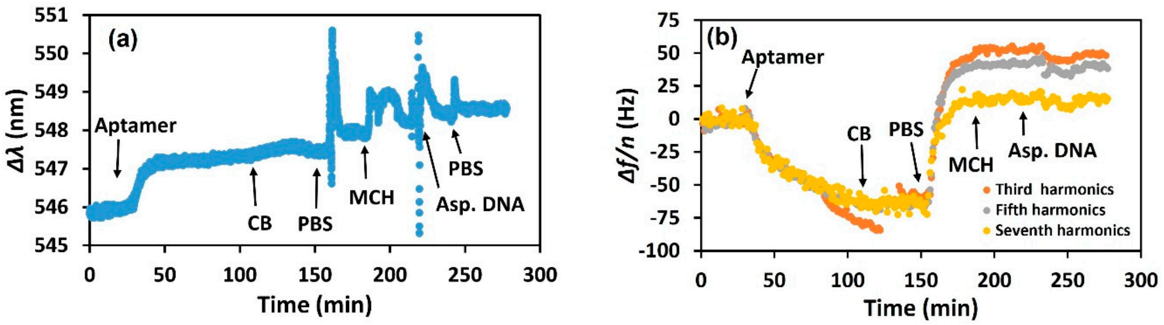

Figure S4. The changes of (a) the wavelength ($\Delta\lambda$) and (b) the normalised resonant frequency ($\Delta f/n$) for 3rd, 5th and 7th overones following addition of aptamer (2 μM), washing the surface by citrate buffer (CB), phosphate-buffered saline (PBS) and following addition of 6-mercapto-1-hexanol (MCH) and salmon sperm DNA. The moments of additions are indicated by arrows.

S5. Comparison of the sensitivity of the aptamer-based biosensors for detection of penicillin G

The comparative analysis of the basic characteristics of the aptamer-based biosensors for penicillin G (PEN) detction is sumamrized in Table S1.

Table S1. The aptamer-based biosensors for penicillin G detection. The constant of dissociation, K_D, and limit of detection (LOD) are shown for buffer.

Aptamer sequence 5'→ 3' and modification	Surface layer	Detection method/probe	K_D, nM	Real sample	Dynamic range, LOD ng/mL	Ref.
**Electrochemical detection**						
**NH$_2$**-(CH$_2$)$_6$-CTG AAT TGG ATC TCT CTT CTT GAG CGA TCT CCA CA	GCE: GR–Fe_3O_4NPs and PE-DOT–AuNPs	DPV/ $[Fe(CN)_6]^{3-/4-}$	-	Milk	0.1-200/ 0.057	[2]
GGG AGG ACG AAG CGG AAC GAG ATG TAG ATG AGG CTC GAT CCG AAT GCG TGA CGT CTA TCG GAA TAC TCG TTT TTA CGC CTC ATA AGA CAC GCC CGA CA	4-NB modified SPCE	EIS/ $[Fe(CN)_6]^{3-/4-}$	-	Milk	0.4-10^3/ 0.17	[3]
**SH**-(CH$_2$)$_6$-TTA GTT GGG GTT CAG TTG G	Pencil graphite electrode modified with rGO-AuNPs	EIS/ $[Fe(CN)_6]^{3-/4-}$	-	Milk	$3.5x10^{-4}$-3.5/ $2.8x10^{-4}$	[4]
**NH$_2$**-(CH$_2$)$_6$-CTG AAT TGG ATC TCT CTT CTT GAG CGA TCT CCA CA	Au electrode covered with Ag based MOF	EIS/ $[Fe(CN)_6]^{3-/4-}$	-	Milk	0.001 – 0.5/ 0.849 10^{-3}	[5]
**HS**-(CH$_2$)$_6$-GGG TCT GAG GAG TGC GCG GTG CCA GTG AGT-**MB**	Au electrode	SWV/MB	-	Milk	-/0.11	[6]
**HS**-(CH$_2$)$_6$-CTG AAT TGG ATC TCT CTT CTT GAG CGA TCT CCA CA	Electrospun carbon nanofiber-AuNPs	LSV/ $[Fe(CN)_6]^{3-/4-}$	-	Milk	1–400/ 0.6	[7]
CTG AAT TGG ATC TCT CTT CTT GAG CGA TCT CCA CA	Au: Carbazole-bearing porous organic polymer	EIS/ $[Fe(CN)_6]^{3-/4-}$		Milk Human serum	0.001-10/ $0.32x10^{-3}$	[8]
**HS**-(CH$_2$)$_6$-GGG TCT GAG GAG TGC GCG GTG CCA GTG AGT TTT TTG GGT CTG AGG AGT GCG CGG TGC CAG TGA GT	Au	EIS/ $[Fe(CN)_6]^{3-/4-}$	-	Tap water	$3.5x10^{-4}$-3.5 /$1.05x10^{-4}$	[9]
**HS**-(CH$_2$)$_6$-TAG GGA ATT CGT CGA CGG ATC CGC GTA GAG GCA TCC TAG GAC GAA CG	GCE: AuNPs-Fe_3O_4-MWCNTs	DPV	8.2	Milk	0.7-$3.5x10^3$/ 0.23	[10]
**HS**-(CH$_2$)$_6$-TAG GGA ATT CGT CGA CGG ATC CGC GTA GAG GCA TCC TAG GAC GAA C-Fc	GCE: Fe-N-C-CNTs - Nb_2C-MB	DPV	-	Milk	-/0.033	[11]
**NH$_2$**-(CH$_2$)$_6$-CTG AAT TGG ATC TCT CTT CTT GAG CGA TCT CCA CA	rGO	DPV	-	Milk	-/$0.43x10^{-3}$	[12]
GGG TCT GAG GAG TGC GCG GTG CCA GTG AGT	GCE: HOF	DPV	-	Milk	$5x10^{-4}$-0.5/ $1.04x10^{-6}$	[13]
**Optical detection**						

**FAM**-GGG TCT GAG GAG TGC GCG GTG CCA GTG AGT	rGO	Fluorescence	383.4		-/3.18	[14]
GGG AGG ACG AAG CGG AAC GAG ATG TAG ATG AGG CTC GAT CCG AAT GCG TGA CGT CTA TCG GAA TAC TCG TTT TTA CGC CTC ATA AGA CAC GCC CGA CA	AuNPs	Colorimetry	-	-	3000-27000/3000	[15]
**Dual mode**						
**HS**-$(CH_2)_6$-CTG AAT TGG ATC TCT CTT CTT GAG CGA TCT CCA CA	AuNPs<br>Au	LSPR<br>QCM-D	20<br>50	-	1.09<br>1.05	This work
**Multi-mode**						
**NH_2-**$(CH_2)_6$-CTG AAT TGG ATC TCT CTT CTT CAG CGA TCT CCA CA	ITO: Zr-UiO-66-NH_2 MOF; Ag-$ZnIn_2S_4$ QDs and magnetic beads separation	PEC, ECL, Fluorescence	-	Milk	3.48x10^{-6}	[16]

4-NB: 4-nitrobenzenediazonium salt; AuNPs: gold nanoparticles; ECL: electrochemiluminescence; EIS: electrochemical impedance spectroscopy; FAM: 6-carboxyfluorescein; Fc: ferrocene; Fe-N-C-CNTs: Fe-N-C doped carbon nanotubes; GCE: glassy carbon electrode; GR–Fe_3O_4NPs: magnetic graphene nanocomposite; HOF: hydrogen-bonded organic frameworks; ITO: indium tin oxide; K_d: dissociation constant; LOD: limit of detection; LSPR: localised surface plasmon resonance; LSW: linear sweep voltammetry MB: methylene blue; MOF: metal organic frameworks; Nb_2C-MB: niobium carbide functionalized with methylene blue; PEDOT: poly(3,4-ethylenedioxythiophene); PEC: photoelectrochemistry; QCM-D: quartz crystal microbalance with dissipation monitoring; QDs: quantum dots; rGO: reduced graphene oxide; SCPE: screen-printed carbon electrode; SWV: square wave voltammetry.

References (References [1–16] are cited also in the main text)

1. Kastner, S.; Dietel, A.-K.; Seier, F.; Ghosh, S.; Weiß, D.; Makarewicz, O.; Csáki, A.; Fritzsche, W. LSPR-based biosensing enables the detection of antimicrobial resistance genes. *Small* **2023**, *19*, 2207953. https://doi.org/10.1002/smll.202207953.
2. Zhao, J.; Guo, W.; Pei, M.; Ding, F. GR–Fe_3O_4 NPs and PEDOT–AuNPs composite based electrochemical aptasensor for the sensitive detection of penicillin. *Anal. Methods* **2016**, *8*, 4391–4397. https://doi.org/10.1039/c6ay00555a.
3. Paniel, N.; Istamboulie, G.; Triki, A.; Lozano, C.; Barthelmebs, L.; Noguer, T. Selection of DNA aptamers against penicillin G using Capture-SELEX for the development of an impedimetric sensor. *Talanta* **2017**, *162*, 232–240. https://doi.org/10.1016/j.talanta.2016.09.058.
4. Mohammad-Razdari, A.; Ghasemi-Varnamkhasti, M.; Izadi, Z.; Ensafi, A.A.; Rostami, S.; Siadat, M. An impedimetric aptasensor for ultrasensitive detection of Penicillin G based on the use of reduced graphene oxide and gold nanoparticles. *Microchim. Acta* **2019**, *186*, 372. https://doi.org/10.1007/s00604-019-3510-x.
5. He, H.; Wang, S.-Q.; Han Z.-Y.; Tian, X.-H. Zhang, W.-W.; Li, C.-P.; Du, M. Construction of electrochemical aptasensors with Ag(I) metal–organic frameworks toward high-efficient detection of ultra-trace penicillin. *Appl. Surf. Sci.* **2020**, *531*, 147342. https://doi.org/10.1016/j.apsusc.2020.147342.
6. Yu, Z.; Cui, P.; Xiang, Y.; Li, B.; Han, X.; Shi W.; Yan, H.; Zhang, G. Developing a fast electrochemical aptasensor method for the quantitative detection of penicillin G residue in milk with high sensitivity and good anti-fouling ability. *Microchem. J.* **2020**, *157*, 105077. https://doi.org/10.1016/j.microc.2020.105077.
7. Vafaye, S.E.; Rahman, A.; Safaeian, S.; Adabi, M. An electrochemical aptasensor based on electrospun carbon nanofber mat and gold nanoparticles for the sensitive detection of penicillin in milk. *J. Food Measur. Charact*. **2021**, *15*, 876-882. https://doi.org/10.1007/s11694-020-00684-x.
8. Yuan, R.R.; He, H.M. Construction of an electrochemical aptasensor based on a carbazole-bearing porous organic polymer for rapid and ultrasensitive detection of penicillin. *Appl. Surf. Sci.* **2021**, *563*, 150307. https://doi.org/10.1016/j.apsusc.2021.150307.

9. Guang, Y.; Xiang, Y.; Yu, Z.; Li, B.; Han, X.; Zhang, Y.; Zhang, G. The binding pattern of the docked two-segment-aptamer to penicillin G and its impedance sensing performance. *Sens. Actuat.: B. Chemical* **2023**, *396*, 134640. https://doi.org/10.1016/j.snb.2023.134640.
10. Hu, M.; Yue, F.; Dong, J.; Tao, C.; Bai, M.; Liu, M.; Zhai, S.; Chen, S.; Liu, W.; Qi, G.; Vrublevsky, I.; Sun, X.; Guo, Y. Screening of broad-spectrum aptamer and development of electrochemical aptasensor for simultaneous detection of penicillin antibiotics in milk. *Talanta* **2024**, *269*, 125508. https://doi.org/10.11016/j.talanta.2023.125508.
11. Hu, M.; Dong, J.; Wang, H.; Huang, J.; Geng, L.; Liu, M.; Tao, C.; Liu, J.; Chen, X.; Ahmed, M.B.M.; Zhao, W.; Sun, X.; Guo, Y. Novel ratiometric electrochemical aptasensor based on broad-spectrum aptamer recognition for simultaneous detection of penicillin antibiotics in milk. *Food Chem*. **2024**, *456*, 139946. https://doi.org/10.1016/j.foodchem.2024.139946.
12. Sahoo, J.; Arya, N.; Gandhi, S. Chemically reduced graphene oxide based assembly of aptasensor for sensitive and probe-free detection of penicillin-G. *Food Chem*. **2025**, *472*, 142914. https://doi.org/10.1016/j.foodchem.2025.142914.
13. Zhao, Y.; Peng, Y.; Liu, Y.; Li, R.; He, H.; Li, C-P.; Wei, C.; Guo, F.; Wei, W. Electrochemical aptasensors based on hydrogen-bonded organic frameworks for detecting trace penicillin G. *Bioelectrochemistry* **2025**, *166*, 109041. https://doi.org/10.1016/j.bioelechem.2025.109041.
14. Lee, A.-Y.; Ha, N.-R.; Jung, I.-P.; Kim, S.-H.; Kim, A.-R.; Yoon, M.-Y. Development of a ssDNA aptamer for detection of residual benzylpenicillin. *Anal. Biochem*. **2017**, 531, 1–7. https://doi.org/10.1016/j.ab.2017.05.013.
15. Darmawati, D.; Mustopa, A.Z.; Budiarto, B.R.; Rahmawati S.I.; Izzati, F.N.; Harmoko, R.; Saepudin, E.; Mahsunah, A.H. *J. Eng. Technol. Sci*. **2022**, *54*, 220413. https://doi.org/10.5614/j.eng.technol.sci.2022.54.4.13.
16. Li, Z.; Wang, B.; Dong, Y.; Jie, G. A multi-modal biosensing platform based on Ag-ZnIn2S4@Ag-Pt nanosignal probe-sensitized UiO-66 for ultra-sensitive detection of penicillin. *Food Chem*. **2024**, *444*, 138665. https://doi.org/10.1016/j.foodchem.2024.138.665.
